# Avoiding a crisis: A national review of implementing the role of advanced practice in radiation therapists in Ireland^☆^

**DOI:** 10.1016/j.tipsro.2025.100355

**Published:** 2025-11-14

**Authors:** Laura Barry, Melanie Clarkson, Erica Bennett

**Affiliations:** a Bon Secours Radiotherapy Cork in partnership with UPMC Hillman Cancer Centre, Cork, Ireland; b College of Health, Wellbeing and Life Sciences, Sheffield Hallam University, United Kingdom

**Keywords:** Advanced practice, Advanced practitioner, Advanced practice radiographer, Radiation therapist

## Abstract

Advanced practice is not a new concept in Irish healthcare, having been established in nursing for decades. However, there is limited clinical career progression for radiation therapists as advanced practice roles have not been formalised within radiotherapy. The same factors driving other jurisdictions worldwide to implement advanced practice roles in radiotherapy are the reasons for change in Ireland today: an ageing population, new technology, recruitment, retention, task-shifting, and optimisation of patient-centred care. A national framework in Ireland has not yet been established.

To identify the key barriers and enablers of implementing advanced practice in radiotherapy in Ireland, a qualitative study was undertaken, which consisted of semi-structured interviews of 10 key senior stakeholders.

The key themes identified were: training/education, professional development, governance and workplace culture. Ethics approval and participant consent were sought in advance.

Participants across both public and private sectors acknowledged the potential of advanced practice in radiation therapy roles to tackle recruitment and retention issues, enhance patient care and optimise service efficiency. Key findings included the need for funded education in addition to frustration around slow implementation despite widespread clinical support.

The recommendations based on the study findings are:

– Creation of an Irish AP implementation steering group
– Create a specific APRT guidance framework document.
– Recognition of master’s level education requirement for the role to be undertaken and completed in post in a candidacy style

## Highlights

• Clinical career progression in radiotherapy in Ireland is limited.
• Many radiation therapists are re-training or leaving healthcare entirely as a result.
• Advanced practice has proven increased efficiencies, retention and job satisfaction.
• Advanced practice is not yet recognised for radiation therapists in Ireland.
• Clarity on education requirements, funding and governance are required.

## Introduction

Advanced practice (AP) in radiotherapy is not a new idea but has existed for over twenty years [1]. In Ireland, a national Health and Social Care Professional Framework was published in 2023 after ten years of development and has yet to be implemented into practice [2]. The same catalysts that prompted other jurisdictions to adopt AP roles in radiotherapy (RT) twenty years ago are driving change in Ireland today: an aging population, expensive new technology, rising costs, recruitment, retention, and the increasing complexity of care, compounded by a shortage of radiation oncologists (R.Os) which intensifies pressure within the system [3,4].

Many European countries are incorporating AP into their RT pathway, with the European Society for Radiotherapy and Oncology (ESTRO) Radiation Therapist (RTT) committee advocating for AP roles to provide professional progression, enhance access to and efficiency of treatment, as well as optimising patient-centric care [5]. As AP in RT is not yet officially recognised in Ireland, the lack of clarity surrounding the scope and governance presents both an implementation and integration challenge. This role has been successfully implemented in other global jurisdictions, such as the United Kingdom (UK) and Canada in particular, with both countries having contributed largely to the international evidence base surrounding implementation, framework, governance and impact [6,7].

Radiotherapy in Ireland, has limited clinical career progression. There is currently a staffing crisis within radiotherapy, with many RTTs retraining in other healthcare disciplines or leaving healthcare entirely due to a lack of progression and fear of stagnation [8]. It is a pivotal moment with the publication of an AP framework for allied health professionals in Ireland, providing the opportunity to ensure patient pathways are more efficient and improve recruitment and retention rates through AP implementation [2].

To further explore the current environment, a qualitative review was undertaken to consider the perceived barriers and enablers of key decision makers within radiotherapy in Ireland in relation to implementing AP roles for RTTs. The intention is for the results of this study to be used to provide recommendations for the development of an AP framework for radiotherapy in Ireland.

## Materials and methods

### Study design

A descriptive phenomenological approach (DPA) was undertaken to support the rationale of identifying and exploring the perceived potential barriers and enablers of the participants within the research population [9]. Specifics of the study design are discussed below.

### Population, sampling and recruitment

The research population consisted of key decision makers within the radiotherapy profession in Ireland, including radiotherapy service managers, R.Os, educators and regulators (consisting of Health and Social Care Professionals Council (CORU) and the Health Information and Quality Authority (HIQA)).

**Fig. 1:**
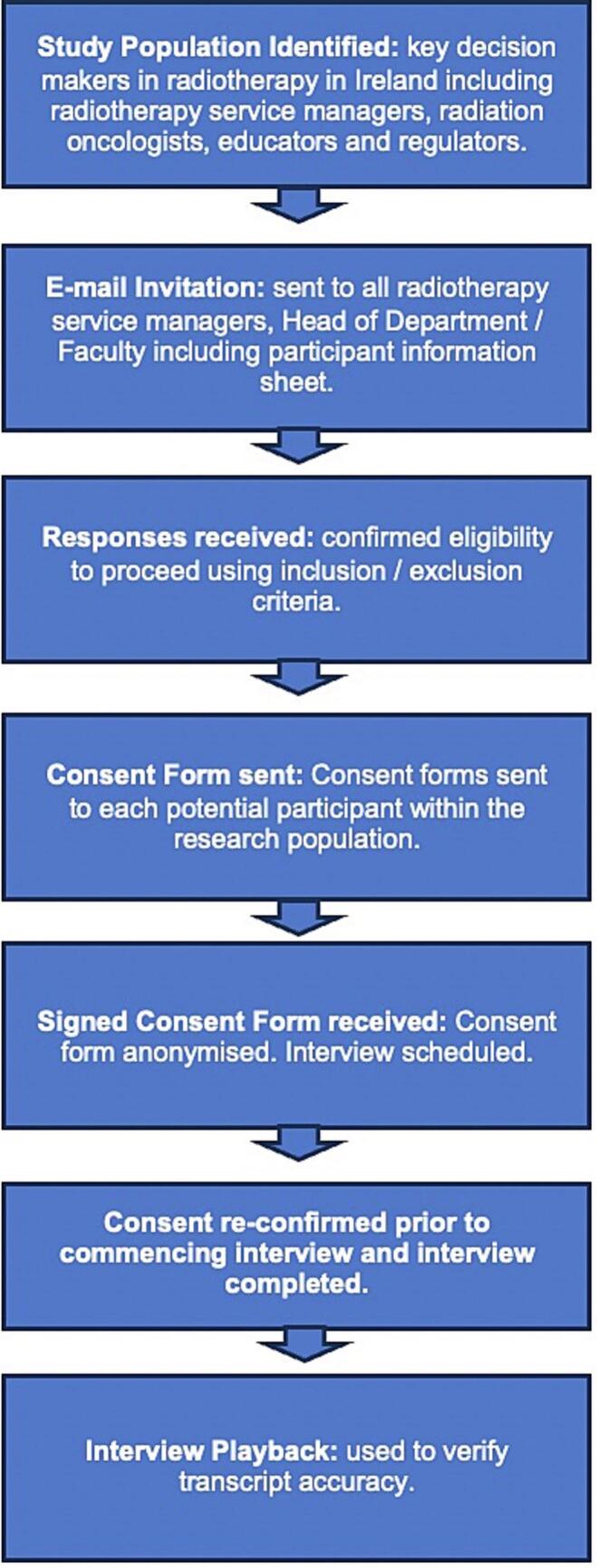
Recruitment schema.

The sampling method used was purposive, as it targeted participants based on their role and experience in relation to the research question [10]. Radiotherapy Managers and R.Os in all radiotherapy departments in Ireland were invited by email to participate. The Radiotherapy Operations Manager of the local department sent this email to an existing group on behalf of the researcher. All Department Heads of universities offering radiotherapy higher education and regulators were contacted through the same method (contact details are publicly available). Snowball sampling occurred through the email, requesting that they forward it to other potentially eligible participants. The recruitment schema is outlined in Fig. 1.

Professionals within the research population were eligible to participate in the study if they met the criteria outlined in Table 1.This study was part of an academic project, aiming to recruit 8–10 participants to provide sufficient qualitative data within the limited timeframe available for the study. Due to the study participants being highly specific to the study aim, the study closed recruitment at 10 participants upon achieving data saturation, providing sufficient information power [11].

**Table 1:** Inclusion and exclusion criteria.

**Inclusion Criteria**	**Exclusion Criteria**
- Those willing to participate, who have given informed consent, including permission to process their data. - Adult professionals in either academia, radiotherapy service management, radiotherapy regulation in Ireland or radiation oncology consultants.	- Not willing to consent. - Anyone not working professionally in either academia, radiotherapy service management, radiotherapy regulation or radiation oncology consultancy. - Radiotherapy students.

### Data collection

Data was collected via semi-structured interviews using Microsoft Teams to facilitate detailed discussion for a deeper exploration of advanced practice radiation therapist (APRT) roles, capturing the diversity of the sample across different stakeholder groups, consistent with a DPA. The same researcher conducted all interviews for consistency, and responses were recorded and transcribed using Microsoft Teams software, verified as correct by the researcher upon playing back the audio recording. The identity of each interviewee was known only to the researcher, with transcripts anonymised to protect the anonymity of the participant.

**Fig. 2:**
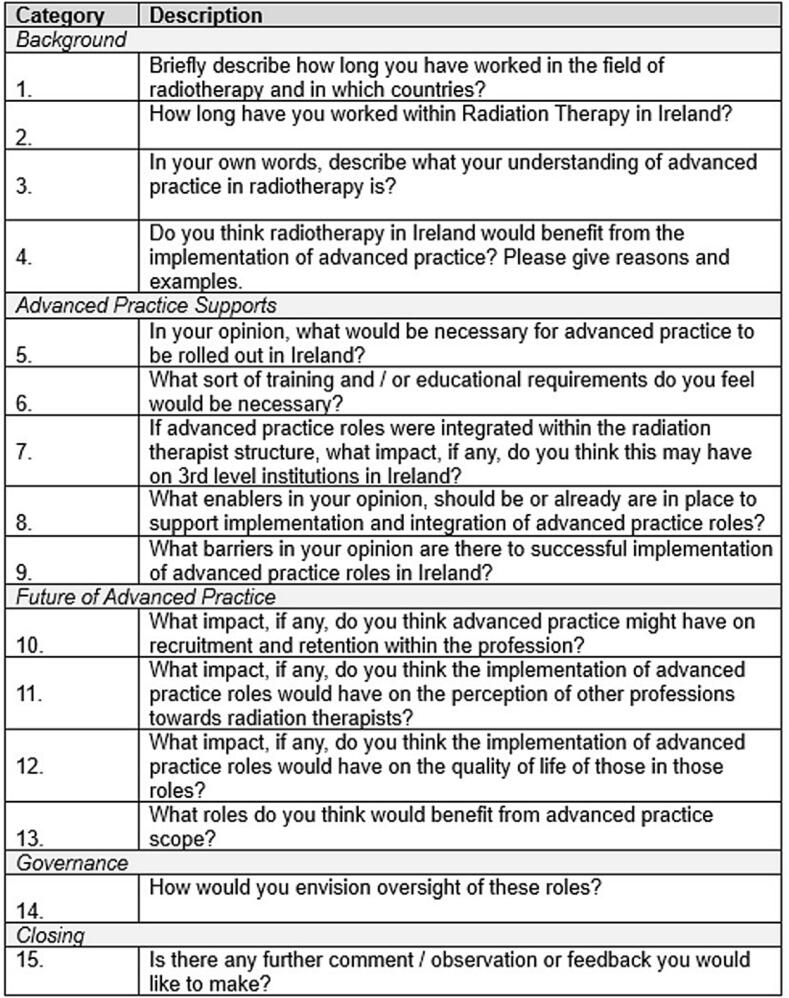
Interview questions.

The interview questions were peer reviewed by two experts: one in advanced practice in radiation therapy and education and the other in radiotherapy service management and healthcare research. The questions were constructed based on a literature review of the research topic undertaken by the researcher, highlighting areas that required further analysis, as shown in Fig. 2 [12].

### Data analysis

DPA with thematic analysis (TA) was utilised to generate a deeper understanding of the participants’ experiences, with TA supporting the review of patterns within their experiences [13]. Semi-structured interviews provide a medium to capture rich descriptions of these experiences, ensuring the dialogue remains focused on answering the research question. TA was used to complement the approach, analysing the transcripts, to identify recurring patterns of meaning related to the research question, helping the researcher explore organisational dynamics in addition to personal experiences. When used together, DPA and TA provide a comprehensive understanding of the phenomenon being studied, identifying meaning from the original data, grouping these into patterns and compiling the results into themes [14]. Rigour was demonstrated by ensuring trustworthiness in data analysis. A different researcher independently coded the transcript data and identified themes. These themes were then discussed and compared against those found by the interviewer, validating the findings and enhancing credibility to reach the final four themes used [15,16]. The codes and themes were generated through an open coding approach. Quotations are presented within the results to illustrate how the data informed the themes, ensuring the findings are grounded in the data [14].

### Ethical considerations

As this research project involved human participants, ethical approval was obtained from both the University and the Hospital. Participant consent was gained before the interview and reconfirmed as the interview commenced (see Fig. 1). There were no withdrawals from the study.

The radiotherapy community in Ireland is small, with the lead researcher working as an RTT, with potential for both parties to be known to each other or to have worked together. It is important to recognise the potential for performance bias in this instance [17]. This familiarity had the potential to influence the interviews; participants may have felt more comfortable sharing their views but equally may have moderated their responses due to the relationship. From the researchers' perspective, prior knowledge of participants’ professional roles and experiences risked influencing the way questions were asked or the interpretation of answers.

Several mitigations were put in place to reduce the impact of bias—the use of a semi-structured interview guide to ensure consistency and to avoid drifting into conversational familiarity. The researcher applied reflexivity principles by recording reflections on the interviews and further engagement with the peer reviewer on the emerging themes.

Although not all biases can be eliminated, these strategies aim to mitigate the risk and ensure transparency and trustworthiness of the research.

## Results

The sample size accrued a geographically diverse population in public and private healthcare sectors, academia, regulators and medical colleagues. The data collection process took place over six weeks, dependent on participant availability, with an average interview duration of 31.2 minutes. The research population distribution is outlined in Table 2.

**Table 2:** Research population distribution.

**Stakeholder Group**	**Number of Participants**
Radiotherapy Management	4
Radiation Oncologists	2
Educators	2
Regulators	2

Thematic analysis of the ten interviews demonstrated four main themes discussed below:

– Education and Training
– Professional Development
– Governance
– Workplace Culture

### Education and training

Management and educator groups felt a master’s degree or a combination of modules was required. In contrast, regulatory and clinician groups showed more uncertainty about the specific requirements.

*‘…I suppose I feel very strongly, yes, that someone who’s an advanced practitioner needs to have a Masters..’ − Educator*

*‘..I see it as a module or a level 9. That’s how I see it.’ – Management*

Largely the participants appeared agnostic around when this education may take place. Several participants referenced advanced nurse practitioners in Ireland, whereby the role is advertised as a candidacy role. Upon successful interview one begins their training on the job, providing equal opportunities and ensures the right person for the job is selected, in addition to relevant education for the role being undertaken. There was some variation amongst the participants in terms of how this might be funded, with willingness to support potential AP trainees, suggesting bonding or co-funding, if funding of the entire course was not possible due to budgetary constraints.

*‘And I do see a lot of parallels with the advanced nurse practitioner system. It’s obviously something that is working quite well and that they start off in the candidate role*…*’ − Regulator*

### Professional development

Dissatisfaction was expressed in relation to the current RTT career pathway in Ireland, with no further opportunity for clinical career advancement beyond the Clinical Specialist role, a role many reach within six to seven years from graduation.

*‘..I think that there is not enough career progression and that if anybody wants to progress, they have to leave the clinical service..’ – Management*

Throughout the interviews, there appeared to be some confusion around the concept of task-shifting, the participants unanimously agreed that the introduction of AP in Ireland would have a positive impact on job satisfaction, recruitment and retention into the profession.

*‘..in terms of attracting other young people, not necessarily young people, but people, new people into the profession, it’s also really important*…*’ – Regulator*

No disadvantages to implementation were outlined throughout the course of the interviews.

### Governance

Supervision was not as prevalent within the interview discussion in comparison to the emphasis placed on mentoring within the literature. It was highlighted particularly by one R.O, who had worked with APRTs previously, that allocating appropriate resources to support AP trainees adequately requires planning, to ensure the potential of the role is reached.

‘*You also need resources to supervise and support these people…Ironically, the team that may benefit the most is a very busy clinical team on the ground, but these people are going to have the least time available to support somebody coming through the service..’ – R.O*

Aspects of governance on both a local and national level in terms of oversight and reporting structure and how this role might be benchmarked and validated nationally, had not been considered yet by many.

*‘..how that works in terms of oversight, I don’t know..’ – R.O*

Both educator and management groups, highlighted funding as a key barrier to those accessing training for APRT and for the remuneration of the post. Despite an appetite to implement APRT, there is an urgent need to gain government approval to secure funding for this role and to facilitate a new grade code and pay scale to reflect this increased level of autonomy and responsibility.

*‘..they would also need to look at the payscale..’ – Management*

### Workplace culture

Workplace culture was a prevalent theme, with many remarking that they felt there was good support from R.Os in particular, with a strong emphasis on buy-in from management and consultants felt to be required for a smooth transition.

*‘…our consultants are very proactive and very forward thinking, so they fully support the development of advanced practice roles for radiation therapists..’ – Management*

The participants all appeared acutely aware of the potential for conflict upon integration of the role within the multidisciplinary team (MDT) and the redistribution of some tasks from one profession to another. The radiotherapy management cohort had quite a pragmatic stance, in not wishing to antagonise or ‘take’ work from another profession but instead appeared resolute in carving out a niche role for APRTs where they would have the knowledge and skills to be best placed to deliver care within that sphere.

An individual’s understanding of what AP is influences how they perceive the role evolving and what shift within expectations might be required. At an operational level there appeared to be a good understanding of what the role would look like within a service, and which aspects might need to be clearly communicated, to negate any conflict.

*‘…how to optimally kind of have those professions all working in tandem and to be able to kind of, you know, have the right skills at the right place…’ − Management*

There was a large focus placed on demonstrating value and how the role might enhance the pathway. This requires the performance review process with managers and APRTs to be goal orientated and motivate staff. Metrics supporting the benefits of AP are needed to support a larger scale implementation and integration of the role.

*‘..our own metrics about, you know, is the person actually fulfilling the role? Are they meeting all the different pillars…’ − Educator*

## Discussion

### Education and training

These results align with the findings of a recent publication, which found that 73 % of their respondents considered a master’s degree the standard [18]. The UK, Australia and New Zealand have outlined that a master’s qualification is required to operate across the four pillars of practice successfully (clinical practice, leadership and management, research and education), and this is likely the route that will be taken in Ireland [19], [20], [21], [22].

The data indicates evidence of the requirement for master’s level education, but that this can be done in post, in a candidacy style role with agreements in place regarding protected study days and funding for education. Otherwise, it is a significant financial and time burden to expect individuals to undertake with no guarantee of a role upon education completion, which could result in limited interest and uptake in the role.

Universities in Ireland suggested collaboration between clinical departments and education providers to ensure the courses available to those in Ireland reflected the AP roles available. Flexibility from providers was cited as important to prevent education from becoming a barrier. Rolling intakes and the possibility of working incrementally towards qualifications would enable AP trainees to continue working and undertake part-time education [18].

### Professional development

There is a noted appetite for increased responsibility and autonomy within the profession. RTTs are already highly skilled, technically trained and well placed to offer more specialised dynamic roles, optimising effectiveness and efficiencies to benefit the patients, the service and the professional development of the staff group [23], [24], [25].

The AP role utilises the concept of task shifting, ultimately a reallocation of tasks amongst the MDT, to facilitate optimal use of an individual’s advanced scope of practice [26]. Shared tasks between an APRT and R.O is one of the many great advantages of the role [27]. Task congruency and concordance has demonstrated high standards of care remain uncompromised, emphasising the level that the tasks are undertaken at, not just the task itself [22], [28], [29].

### Governance

Ireland is in a fortunate position that there is a multi-professional framework, which provides a structure for the roll out of AP, with enthusiasm from the management cohort to take ownership of progressing AP [8], [30].

As this is an AP framework for all allied health professionals, there are some aspects that are generic. Additional guidance documentation around job descriptions, educational requirements, funding, regulation pathways and legislative amendments have been recommended to enable success for this type of organisational change [21], [30]. The regulations within Ireland have demonstrated support of AP, through RTTs having both practitioner and referrer status [31].

Although mentorship can be provided from any professional within the MDT, typically the R.O assumes the role of clinical supervisor [30]. This is an important element to structured, successful implementation and integration by ensuring practitioners are supported, supervised and trained as well as a means of accountability in the absence of formal education programmes [32]. There is a risk that by not placing appropriate emphasis on mentoring in the ‘start-up’ phase of this implementation in Ireland, that APRTs may not feel sufficiently supported, which may in turn impact the success and integration of the role. The uncertainty regarding oversight reported in this study highlights that this is an area to be addressed.

Local and national governance structures require careful consideration to ensure that there is a structured pathway to support those looking to develop in APRT roles, whilst safeguarding patients. Individuals operating within this advanced capacity must have the appropriate knowledge, skills and training to do so and have been validated to practice at this level [30]. Despite the importance surrounding validation it is imperative that the chosen means of validation does not become a barrier to those seeking to obtain these roles, requiring a careful balance to be struck [21], [22].

Appropriate remuneration has been identified within the literature as a barrier to those seeking to progress into APRT roles, with many not reporting the increased responsibility as a barrier but expressing the requirement to be remunerated accordingly. The research participant views were consistent with these opinions [18], [33], [34].

### Workplace culture

This potential for conflict around integration into the service, has been well documented across many jurisdictions [28], [35]. Open communication across all professions, at all levels, (particularly when there is a perception of roles overlapping) can be a strategy to diffuse potential professional tension and territorialism [20], [30]. Strong support and buy in from management and R.Os are identified as essential for smooth implementation and integration [36].

There is no current standardised job description for an APRT due to each country using the role for different functions, which is a challenge for countries such as Ireland who are endeavouring to implement the role [37]. AP development should be responsive to service demands, with the intention of this being planned in a more considered, pro-active manner being led by strategic priority instead of reacting to operational demands [29]. This may explain why at a strategic level there appeared to be a disconnect in terms of what AP is and what that might look like. This is reflective of international experience where there is a lot of confusion due to different titles, educational requirements and responsibilities across Europe alone, making it difficult to differentiate and derive a clear and cohesive matrix of title, responsibility and educational requirement [23], [38].

It is important that Ireland endeavours to communicate through the appropriate channels to government bodies. Successful implementation of AP roles in Australia was hindered without clear leadership strategies, positive professional relationships or recognition of the role [21]. Australia launched their APRT programme at a similar time to the U.K.'s launch of the four-tier structure. Now, in Australia, there are only 2 formally recognised APRTs [30]. This serves as a stark warning of the importance of cohesive roll out and recognition for Ireland.

## Conclusion

This is the first qualitative study in relation to APRT implementation in Ireland, providing the Irish radiotherapy community with the opportunity to contribute to the national and international evidence base on the topic. The researcher appreciates that there is some unavoidable bias, as the participants that volunteered had an invested interest in the topic. However, this highlights that stakeholders within the profession want the opportunity to be heard.

To drive the initiative forward, the key recommendation from this research is that an Irish AP implementation steering group is required, representing the key stakeholders, chaired by a radiotherapy service manager(s), to centralise efforts of APRT progress in a united manner. Although government support and buy-in is essential, individuals with operational insight must be involved in these discussions. The second recommendation is that an APRT-specific guidance document (to supplement the current multidisciplinary AP framework) is required to address the unique aspects of the APRT role. Clarification on master’s level education requirements and funding, reporting structure, job descriptions, mentoring, oversight and credentialing would provide a structured framework pathway for RTTs specifically. In addition, clear training pathways, core capabilities and goals are needed to facilitate a successful implementation and integration of this new role [8], [30], [38].

## Informed patient consent

The authors confirm that written informed consent has been obtained from the involved participants; and, they have given approval for this information to be published in this case report (series).

## Declaration of competing interest

The authors declare that they have no known competing financial interests or personal relationships that could have appeared to influence the work reported in this paper.
